# A comparison of the Dobbs method for correction of idiopathic and teratological congenital vertical talus

**DOI:** 10.1007/s11832-016-0727-7

**Published:** 2016-04-02

**Authors:** Yuen Chan, Veenesh Selvaratnam, Neeraj Garg

**Affiliations:** Alder Hey Children’s NHS Trust, Eaton Road, Liverpool, Merseyside L12 2AP UK

**Keywords:** Congenital vertical talus, Dobbs method, Idiopathic, Teratological

## Abstract

**Introduction:**

Congenital vertical talus (CVT) is a rare deformity. Traditionally, CVT correction involved extensive soft tissue releases, and this was associated with high complication rates. The Dobbs method is less invasive and comprises serial manipulation and casting, followed by minimally invasive reduction and K-wire fixation of the talonavicular joint and percutaneous Achilles tenotomy.

**Aim:**

The purpose of our study was to compare the outcomes of using the Dobbs method for CVT correction in idiopathic and teratological patients.

**Methods:**

A retrospective analysis of all patients treated with the Dobbs method for CVT between the years 2007 and 2012 was carried out. Notes, electronic records and radiographs were reviewed for every patient. The Oxford ankle foot score was obtained at follow-up.

**Results:**

There was a total of ten children with 18 affected feet. Five children (ten feet) had teratological CVT. Recurrence was noted in six feet (two from the idiopathic group and four from the teratological group). The median age was 5 months (range 2–8 months). The mean follow-up was 53 months (range 24–80 months). There was a significant difference between pre-operative to initial post-operative and pre-operative to latest follow-up measurements for all angles in the successfully treated CVT group (*p* < 0.000001). The mean Oxford ankle foot scores for each domain in all patients are 59.9 (physical), 88.8 (school and play) and 99.1 (emotional).

**Conclusion:**

The Dobbs method is a less invasive technique of CVT correction. It is an effective initial method of treatment in both teratological and idiopathic patients. A higher recurrence rate was observed in the teratological group, although this was not statistically significant.

## Introduction

Congenital vertical talus (CVT) is a rare deformity in paediatric patients. It consists of dislocation of the talonavicular joint with the navicular dorsally displaced on the talus. The hindfoot is in marked equinus and valgus, and the forefoot is abducted and dorsiflexed at the midtarsal joint [1]. The incidence of CVT is 1/10,000 births. In 50 % of the cases, it is associated with an underlying neuromuscular or genetic condition [1, 2]. In some patients, there may be a genetic component to this condition. There is increasing evidence to show an autosomal dominant inheritance pattern in families [3–5]. There is a positive family history in 12–20 % of cases [2].

Correction of CVT traditionally involved extensive soft tissue releases to allow restoration of the normal anatomical relationships between the bony structures within the foot. Casting was used to improve the deformity prior to carrying out extensive surgery [1]. These extensive surgical procedures were associated with high rates of complications, including severe stiffness of the ankle and subtalar joint, avascular necrosis of the talus, undercorrection of the deformity and recurrence [6–10].

There has been a shift to using the Dobbs method in CVT correction. It is a simpler method and easier to apply in young infants. The Dobbs method comprises serial manipulation and casting, followed by minimally invasive reduction and K-wire fixation of the talonavicular joint and percutaneous Achilles tenotomy [11]. The results from this method of correction in both idiopathic and those with associated neuromuscular or genetic conditions have been excellent [12, 13]. As the Dobbs method is a less invasive approach, it appears to avoid the risks associated with more extensive soft tissue releases [13, 14].

Only a few papers have looked at the Dobbs method for CVT correction in both idiopathic and teratological patients [14–16]. Previous papers have looked at patients with idiopathic deformity or patients with associated neuromuscular or genetic disorders only as a single group [13, 17, 18]. The Dobbs method can be more challenging in patients with associated neuromuscular or genetic conditions [13]. The purpose of our paper is to compare the outcomes of using the Dobbs method of serial manipulation, casting and limited surgery for CVT correction in idiopathic and teratological patients.

## Methods

This is a retrospective analysis of all patients treated with the Dobbs method for CVT between the years 2007 and 2012. All patients at our institution over the study period with CVT was treated with the Dobbs method as described by Dobbs et al. [11] A total of ten children with 18 affected feet (nine right feet and nine left feet) underwent the Dobbs method for correction of their CVT. Five children (ten feet) had teratological CVT. Eight feet had arthrogryposis and two had myelomeningocele. Case notes, electronic records and radiographs were reviewed for every patient. On the anteroposterior (AP) radiographs, measurements were taken for the talocalcaneal angle and talus-first metatarsal angle. On the lateral radiographs, measurements were taken for the talocalcaneal angle, talus-first metatarsal angle and tibiocalcaneal angle. All angles were measured on radiographs taken pre-operatively, immediately post-operatively and at the latest follow-up.

**Fig. 1:**
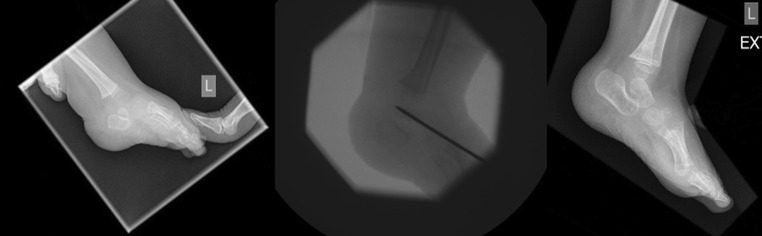
Pre-operative, intra-operative and post-operative images of a child with a left-sided congenital vertical talus (CVT) treated with the Dobbs method

Diagnosis of CVT was according to the Hamanishi criteria with lateral radiographs of the foot in full plantar flexion and dorsiflexion. In maximum plantar flexion, a talar axis-first metatarsal angle >30° is diagnostic of CVT. An increased tibiocalcaneal angle on the lateral radiograph in maximum dorsiflexion would indicate a fixed equinus contracture of the hindfoot [19]. All patients were treated using the standard Dobbs method to correct their CVT deformity (Fig. 1) [11, 12, 20].

The treatment begins with the use of serial manipulations and castings as described by Dobbs [11, 12, 20]. Once the correction is achieved by measurements of a lateral talar axis-first metatarsal angle in maximal plantarflexion of <30°, the child undergoes percutaneous talonavicular joint pinning and percutaneous Achilles tenotomy. Post-surgery, a long leg cast was used for 5–6 weeks, changed every 2 weeks. The patient then uses a bar and brace system full time for 2 months, followed by night time use for 2 years. If the patient is old enough to walk, a solid ankle–foot orthosis is used in place of the bar and brace system [11, 12, 20].

Routine follow-up was in the operating surgeon’s orthopaedic clinic, which consisted of a clinical assessment of the foot and ankle, an assessment of the cosmetic appearance of the foot and any abnormal shoe wear. Radiographs were taken at follow-up and the radiographic angles were measured independently by VS. At the latest follow-up, all patients’ parents completed an Oxford ankle foot score. The Oxford ankle foot score is a 15-item questionnaire scaled on a score of 0–4 per item (0 is always and 4 is never); therefore, a higher score indicates a better outcome. Items 1–14 are split into three domains looking at physical, school and play, and emotional domains. Item 15 is a separate part about footwear [21].

### Statistical analysis

One-way analysis of variance (ANOVA) was performed to measure the differences between the pre-operative, post-operative and latest follow-up angles measured on radiographs. Post hoc analysis was carried out using Student’s *t* test. Fisher’s exact test was used to analyse categorical data and the Mann–Whitney *U* test was used to analyse ordinal data. Pearson’s correlation coefficient is performed for correlation. A *p*-value of less than 0.05 was considered significant. The analysis was carried out using IBM SPSS Statistics 23.

## Results

There were seven boys and three girls with eight idiopathic and ten teratological feet. The median age was 5 months (range 2–8 months) and the mean number of castings was 9 (range 8–10 castings). The mean follow-up was 53 months (range 24–80 months). Recurrence was noted in six feet, two from the idiopathic group and four from the teratological group. The difference in the rate of recurrence between the two groups was not statistically significant (*p* = 0.64, Fisher’s exact test).

The radiographic parameters at the time of the latest follow-up were compared with those recorded pre-operatively and immediately post-operatively. There is a significant difference between pre-operative to initial post-operative and pre-operative to latest follow-up for all angles in the successfully treated CVT group (*p* < 0.000001) (Table 1).

**Table 1:** Mean radiological measurements pre-operatively, early post-operatively and at the latest follow-up for successfully treated congenital vertical talus (CVT) with the Dobbs method (all angles shown as mean ± standard deviation, SD)

Measurement	Pre-op	Initial post-op	Last follow-up	*p*-Value (one-way ANOVA)
AP talocalcaneal	55.8 ± 7.8	19.4 ± 3.5	21.2 ± 4.9	<0.000001*
AP talar-first metatarsal	36.9 ± 10.6	7.4 ± 3.3	9.7 ± 6.7	<0.000001*
Lateral talar-first metatarsal	47 ± 14.5	7.8 ± 6.1	10.2 ± 6.2	<0.000001*
Lateral tibiocalcaneal	104.8 ± 10.1	77.8 ± 7.1	83.4 ± 5.5	<0.000001*
Lateral talocalcaneal	60.4 ± 9.1	31.8 ± 9.7	33.5 ± 9.4	<0.000001*

In the group with recurrence, there were no significant differences between the pre-operative, post-operative and latest follow-up results in the AP talocalcaneal and lateral talocalcaneal angles (Table 2). There was a significant difference between the pre-operative, immediately post-operative and latest follow-up angles in AP talar-first metatarsal angle (one-way ANOVA). Post hoc analysis did reveal a significant difference. There was a significant difference in the lateral talar-first metatarsal angles (one-way ANOVA). Post hoc analysis showed a significant difference between the pre-operative and immediately post-operative angles (*p* = 0.005) but no significant difference between pre-operative and latest follow-up angles (*p* = 0.30). There was a significant difference in the lateral tibiocalcaneal angles (one-way ANOVA). Post hoc analysis showed a significant difference between pre-operative and immediately post-operative angles (*p* = 0.001) and no significant difference between pre-operative and latest follow-up angles (*p* = 0.07).

**Table 2:** Mean radiological measurements pre-operatively, early post-operatively and at the latest follow-up for patients with recurrence (all angles shown as mean ± standard deviation, SD)

Measurement	Pre-op	Initial post-op	Last follow-up	*p*-Value (one-way ANOVA)
AP talocalcaneal	54.9 ± 0.1	40.2 ± 11.9	46.8 ± 9.1	0.25
AP talar-first metatarsal	72.6 ± 2.3	27 ± 20.0	46 ± 10.8	0.02*
Lateral talar-first metatarsal	52.2 ± 22.0	23 ± 9.2	52 ± 16.4	0.02*
Lateral tibiocalcaneal	109 ± 8.5	84 ± 8.3	100.8 ± 3.4	0.003*
Lateral talocalcaneal	63.9 ± 12.3	42.6 ± 11.5	52.6 ± 8.1	0.05

The lateral talocalcaneal angle and lateral tibiocalcaneal angle, which indicates a correction of hindfoot equinus, were significantly smaller post-treatment in those successfully treated using the Dobbs method (*p* < 0.000001). In those who had a recurrence, the lateral tibiocalcaneal angle was significant (*p* < 0.02) only between pre-operative to immediately post-operative measurements. There was no significant difference when compared to the latest follow-up measurements. There was no significance in the lateral talocalcaneal angle. There were significant reductions in the lateral talar-first metatarsal angle in both groups (successful, *p* < 0.000001, recurrence; *p* < 0.02), indicating a correction of the talonavicular dislocation. There were significant reductions in the AP talar axis-first metatarsal angle in both groups (successful, *p* < 0.000001; recurrence, *p* < 0.02), reflecting the correction of the abduction deformity. The AP talocalcaneal angle was significant in the successfully treated CVT, which shows a correction of the valgus deformity (*p* < 0.000001).

**Fig. 2:**
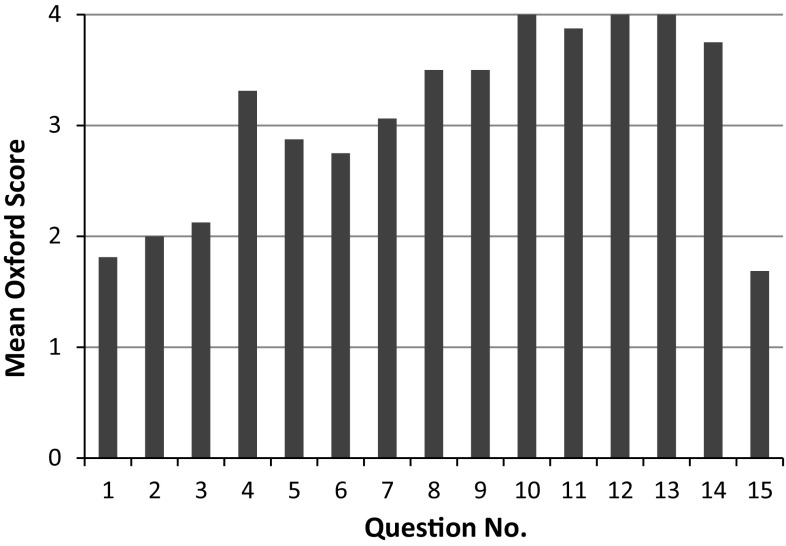
Graph showing the mean score per question for all patients who completed the Oxford foot ankle score

The mean Oxford ankle foot scores for each domain in all patients was 59.9 (physical), 88.8 (school and play) and 99.1 (emotional). The mean score for each question is illustrated in Fig. 2. The mean score in the physical domain was 41.09 in teratological patients and 82.81 in idiopathic patients. The mean score in the school and play domain was 76.56 in the teratological group and 97.66 in the idiopathic group. The emotional domain score was 96.88 in the teratological group and 98.44 in the idiopathic group. There was a significant difference between the two groups in the physical domain (*p* = 0.002, Mann–Whitney *U* test) and the school and play domain (*p* = 0.004, Mann–Whitney *U* test). There was no significant difference between the two groups in the emotional domain (*p* = 0.71).

The lateral talar-first metatarsal angles measured at the latest follow-up showed a medium negative correlation with the physical domain (Pearson correlation coefficient = −0.41, *p* = 0.169) and a small negative correlation with the school domain (Pearson correlation coefficient = −0.14, *p* = 0.640). There was a small positive correlation with the emotion domain (Pearson correlation coefficient = 0.19, *p* = 0.509).

## Discussion

CVT is a rare rigid flatfoot disorder. Half of the cases has an association with teratological disorders [1]. Patients with teratological CVT are associated with poorer outcomes. If left untreated, CVT talus can cause significant long-term disability. The child will experience issues with gait, balance, pain and difficulties with footwear [3]. The goal of the treatment is to restore the normal alignment of the talus, navicular and calcaneal bones. Casting alone was associated with poor outcomes and this led to surgery being the treatment of choice [22]. Surgery included large soft tissue releases due to the multiple deformities involving contractures of the Achilles tendon, peroneus tertius, long toe extensors and tibialis anterior. Patients often undergo several intensive surgeries and may need a subtalar arthrodesis at a young age. Surgery causes long-term complications such as severe stiffness, avascular necrosis of the talus, under-/overcorrection of the deformity and recurrence [6–10].

Ponseti revolutionised the treatment of clubfoot and reduced the need for extensive open surgery [23]. Dobbs described the reverse Ponseti method using serial manipulation and casting, along with minimally invasive surgery to correct CVT. Many studies have shown excellent results using the Dobbs method [12–15]. Dobbs looked at 19 feet in 11 children, all with idiopathic CVT. They found all patients to have excellent to good results in clinical appearance, function, deformity correction and radiographic measurements [12]. Another study looking at just idiopathic feet in four children (four feet) found good results using the reverse Ponseti method. The study found a reduction in both the mean talocalcaneal angle and the mean talar axis-first metatarsal base angle before and after casting. No recurrence was noted; however, the mean follow-up in this study was only 8.5 months [17]. Using the Dobbs method may lead to more flexible and functional feet such as the Ponseti method did for clubfeet. The Dobbs method does this by avoiding the need for extensive soft tissue release procedures [16, 20].

It has been suggested that CVT associated with teratological feet is due to muscle imbalance, which may be a slightly different pathology to idiopathic CVT. Weak or absent motor function in the lower leg is predictive of poor response to treatment and relapse [20]. However, muscle abnormalities may also be a factor in idiopathic cases [24]. Although Dobbs initially described the use in idiopathic CVT, the Dobbs method has been successfully used in the treatment of the extremely rigid vertical talus associated with neuromuscular and genetic syndromes [13–16, 25]. Yang and Dobbs compared minimally invasive and extensive soft tissue releases in the treatment of CVT for both idiopathic and teratological patients with a mean follow-up of 7 years. They found that the minimally invasive treatment method gave better ankle range of movement and pain scores than those treated with extensive surgery and, thus, preserving the mobility and function of the patient’s feet [16]. Another study found excellent to good results in 14 out of 15 patients. They looked at 25 feet altogether, all with teratological CVT. All radiological parameters were significant at the latest follow-up compared with pre-operative parameters (*p* < 0.0001). They had recurrences in 5 out of the 25 feet [13]. A further study looked at both teratological and idiopathic patients. The authors found all radiological parameters to be significant. They reported no recurrences. All feet at follow-up were pain-free and able to plantigrade [15]. Wright et al. also looked at idiopathic and teratological CVT and found it to be effective in the initial correction of CVT. They had significant improvement in all measured radiological parameters [14]. Miller and Dobbs recommend the use of the Dobbs method for all vertical tali, including those with severe rigid deformity. They argue that, even in cases where complete correction is not achieved with casting, the definitive surgery required is still less invasive than traditional methods [20].

Our improvements in radiologically measured angles pre-operatively, immediately post-operatively and on latest follow-up are comparable to other studies [12, 14]. When we analysed the successfully treated patients and the patients with recurrence separately, we found a significant reduction in all angles for the successfully treated group between the pre-operative and initial post-operative measurements, as well as the pre-operative and the latest follow-up measurements. However, in the group of patients with recurrence, only 3 of the 5 measured angles comparing pre-operative with initial post-operative results were significant. The initial reduction in measurements may be an indicator of recurrence. More research will need to be carried out in this area to confirm any relationship between the initial reduction in angles and prognosis.

One study looked at using radiological parameters to predict the success of the Dobbs method. They looked at the talar axis-first metatarsal angle in idiopathic and non-idiopathic CVT cases treated with the Dobbs method and found that 14 of the 20 feet were successfully treated. Of the 14 feet that were successfully treated, five were teratological and nine were idiopathic. Six out of the 20 feet had recurrences. They looked at the difference in the pre-operative talar axis-first metatarsal angle in neutral and plantarflexion as an indicator of talonavicular complex mobility. They found the talar axis-first metatarsal angle to be significantly smaller in the unsuccessfully treated group compared with those who were successfully treated (*p* < 0.0001). The authors found that the flexibility of the talonavicular joint could be a predictor of success in minimally invasive treatment [26].

In our study, we had a similar recurrence rate to the original paper by Dobbs et al. [12]. We had a recurrence in six feet, of which four were from the teratological group and two were from the idiopathic group. The recurrence rate ranges from 0 to 47.6 % in the idiopathic group [12–14]. Of note, the study which reported no recurrence had a very short follow-up time. Four of the te teratological feet had recurrence, which is lower than other reports of up to 66.7 % recurrence in the teratological group [14, 26]. There was no significant difference between the rate of recurrence between the teratological and idiopathic patients. This may be due to the small sample size. However, due to the rarity of the disorder, it is difficult to obtain high numbers. The rate of recurrence is higher in the teratological group compared with the idiopathic group. From a recent study, the authors recommended treatment of recurrences with the principles of minimally invasive methods. This again aims to minimise the amount of surgery and maintains the flexibility of the foot [16].

We also looked at outcome measures using the Oxford ankle foot questionnaire for children. We found a significant difference between the teratological and idiopathic groups in the physical domain (*p* = 0.002, Mann–Whitney *U* test) and the school and play domain (*p* = 0.004, Mann–Whitney *U* test). Emotionally, both groups did well. The significant difference in the physical and school and play domains may represent the difficulties with correction of the teratological feet. We did experience higher rates of recurrence with the teratological group. Outcome measures are difficult in young children. Our surveys were completed by the parents, so there is an element of the score being the parent’s opinion of how the child feels. Other studies used outcome scores which combine clinical appearance and radiological parameters to allocate a score; however, none of the scoring systems have been validated [14, 22]. Another issue with the outcome scoring is the underlying diagnosis of associated condition in teratological CVT. It is difficult to ascertain whether the difference in scores is solely due to the patient’s feet symptoms or their overall symptoms from their genetic or neuromuscular disorder. We found a negative correlation between the latest lateral talar-first metatarsal angles and the physical/school domain, meaning the smaller the angles measured, the better the outcome scores. A negative correlation would be the expected outcome. We also found a small positive correlation with the emotion domain. However, due to our small samples, none of these correlations was significant.

There are limitations to our study. Our study is retrospective and, due to the rarity of CVT, we have a small sample size. Another limitation of our study is not having pre-operative outcome scores to compare the improvement in outcome scoring. It would be difficult to obtain a large sample size for CVT patients, and, in the future, prospective multi-centred observational studies will be the only way to increase the sample size, which can help identify the causes and contributing factors to CVT, as well as observing the outcomes from the treatment of CVT. It would enable us to explore any improvement in outcome scoring, as well as whether there is any correlation between the amount of radiological correction and outcome scoring. It would also help recruit an adequate sample size to enable the detection of any statistical significance in areas such as recurrence rate between teratological and idiopathic patients. Currently, there is literature on short- to medium-term outcomes, but whether the benefits seen in these studies translate into adulthood needs to be explored.

The Dobbs method is a less invasive technique of CVT correction and is a useful treatment in both teratological and idiopathic patients. Using the Dobbs method of serial casting and manipulation with minimally invasive surgery gives favourable results and allows the maintenance of a mobile foot. Although not statistically significant, a higher recurrence rate was observed in the teratological group. We would recommend the use of the Dobbs method in the treatment of CVT in both idiopathic and teratological patients.
